# Genetic diversity and genetic structure of *Decalobanthus boisianus* in Hainan Island, China

**DOI:** 10.1002/ece3.5127

**Published:** 2019-04-18

**Authors:** Huan Jiang, Wenxing Long, Hui Zhang, Chengneng Mi, Tao Zhou, Zongzhu Chen

**Affiliations:** ^1^ Institute of Tropical Agriculture and Forestry Hainan University Haikou China; ^2^ Wuzhishan National Long Term Forest Ecosystem Research Station Hainan China; ^3^ Institute of Forestry Science of Hainan Province Haikou China

**Keywords:** gene flow, genetic diversity and structure, human disturbance intensity, sexual and clonal reproduction

## Abstract

*Decalobanthus boisianus *is a native plant of Hainan Island, China, which has caused considerable damage to tropical forest ecosystems in recent decades. Understanding the genetic diversity and structure of this species can facilitate uncovering the molecular mechanism of its invasive ability. Here, we collected 77 individuals of *D. boisianus* spanning 8 distribution areas with a gradient of human disturbance intensity (i.e., low, moderate, and high disturbance intensity groups) to assess patterns of genetic diversity and structure using inter simple sequence repeat (ISSR) markers. We found that a total of 220 loci were scored with 13 primers using ISSR methods, and that 198 loci were polymorphic. The genetic diversity of *D. boisianus* among these eight forests decreased with increasing human disturbance intensity. Over 70% of the total genetic variation was present within populations, while less than 30% of variation was found among populations. There was a high gene flow (1.27) among them due to a lack of effective geographic barriers. The mean Nei's genetic distance of *D. boisianus* populations was found to be relatively small (i.e., 0.07), and the average genetic similarity of the eight populations was high (i.e., 0.93). Our findings indicate that the genetic diversity of *D. boisianus *correlated to human disturbance density, and that *D. boisianus *populations in Hainan Island have frequent gene exchange. We suggest that reduce deforestation to decrease human disturbance may be a good way to prevent the invasion of *D. boisianus*.

## 1 INTRODUCTION

Invasive species are a major problem across the globe and pose a multitude of threats to natural ecosystems (Carboneras et al., 2017). Despite massive efforts to understand the factors influencing invasion potential of introduced species, we still lack a predictive framework for determining which species are most likely to become invasive. This may be due to the fact that most research on this topic has focused on life‐history traits and not on genetic diversity. Recently, many studies have demonstrated that genetic diversity of invasive plant species leads to their strong ability to adapt to environmental change and facilitates their capacity to spread to novel habitats (Bozkurt, Muth, Parzies, & Haussmann, 2015; Prentis, Wilson, Dormontt, Richardson, & Lowe, 2008; Templeton, 1994). Therefore, it is essential to investigate the genetic diversity and structures of invasive plants to better understand how these plants successfully invade and cause ecological damages.

Environmental pressure is seen as an important factor accounting for plant reproductive strategies, which can further influence levels of genetic diversity in the species (Cook, 1985; Eriksson, 1997; Philbrick & Les, 1996; Sinclair et al., 2016). For example, sexual reproduction typically increases genetic diversity in a population. In contrast, clonal reproduction allows species to rapidly colonize available habitats, but often leads to a decline in genetic diversity (Ellstrand & Roose, 1987; Hamrick, Linhart, & Mitton, 1979; Schmid, Bazzaz, & Weiner, 1995). Several studies have showed that environmental pressures often have negative consequences for genetic diversity. Rusterholz, Kissling, and Baur (2009) demonstrated that soil disturbance induced by human trampling reduced the frequency of sexual reproduction for *Anemone nemorosa* populations, resulting in lower levels of genetic diversity for these populations. Likewise, Aveliina et al. (2009) demonstrated that the genetic diversity of *Briza media* was negatively associated with environmental pressures caused by high population densities of humans.

*Decalobanthus boisianus* (Gagnep.) A. R. Simões & Staples is a liana that distributed in Hainan Island and Guangzhou City. It also has three varieties including *Decalobanthus boisianu*s *var. boisiana, Decalobanthus boisianus var. fulvopilosa, *and *Decalobanthus boisianus var. sumatrana*, which distributed in Yunan and Guangxi Province of China, as well as Laos, Vietnam, Malaysia, Kalimantan Island, and Sumatra Island (Flora of China Editorial board, 1995; Wang et al., 2005; Wang, Peng, Li, & Zhou, 2009; Staples, 2010). This species has recently moved from genus *Merremia* Hall. f. to *Decalobanthus* Ooststr since the former now only included polyphyletic species because of the weak support for monophyly and strong support for polyphyly of this genus (Simões & Staples, 2017). Therefore, *D. boisianus* is more closely related to species such as *Decalobanthus peltatus* (L.) A. R. Simões & Staples which is also a harmful weeds, *Decalobanthus borneensis* (Merr.) Simões & Staples, *Decalobanthus eberhardtii *(Gagnep.) Simões & Staples, and *Decalobanthus pacificus* (Ooststr.) Simões & Staples, ect. *D. boisianus *is adapted to a wide range of ecological conditions, due to its ability to undergo both sexual and clonal reproduction (Lian et al., 2007; Liu, 2007; Wang et al., 2009; Wu, Liang, Chen, Li, & Cao, 2007). A number of studies assumed that viable seeds of the species can be transported over streams (Wu et al., 2007; Wang et al., 2009), which helps *D. boisianus* spread long distance. The species is thus characterized by rapid growth and a strong competitive ability (Huang et al., 2015), which allow it to easily invade plantation areas, shrublands, and secondary forests with low canopy density (Wang et al., 2009; Huang et al., 2015), covering more than 2000 ha areas in Hainan Island. After invasion, it often kills a large number of trees and understory plants with low canopy coverage (Huang et al., 2013). Although researchers suggest that *D. boisianus* can be taken advantage and used for vegetation recovery in barren mountains, as well as leaves feeding livestocks (Sun, Shen, Wan, & Xie, 2006), it has been listed as a major forest harmful pest in China due to the serious damage to secondary forests that the species causes (State Forestry Administration, China).

In this study, we explored genetic diversity and structures of *D. boisianus* in eight Nature Reserves in Hainan Island. According to field surveys and interviews with local residents, we found that *D. boisianus* distributed in areas with relatively high human disturbance intensity. Human disturbance intensity has been proved to affect genetic diversity of plant species (Aveliina et al., 2009; Ledo & Schnitzer, 2014; Rusterholz et al., 2009). Increases in human disturbance intensity across the eight distributed areas of *D. boisianus* likely led to a decrease in its genetic diversity. We thus hypothesized that genetic diversity of *D. boisianus *was high in areas with the low human disturbance intensity, and vice versa.

## 2 MATERIALS AND METHODS

### 2.1 Sample collection

Samples of *D. boisianus *were collected from eight forest sites in Hainan Island. A total of 8–10 individuals were randomly selected in each forest site, with at least 100 m intervals between adjacent individuals to reduce possibility of sampling clones. For each individual, three to four fresh leaves were collected and placed in a zip‐lock plastic bag with 20 g of silica gel. In total, 77 individuals from the eight populations were collected.

**Figure 1:**
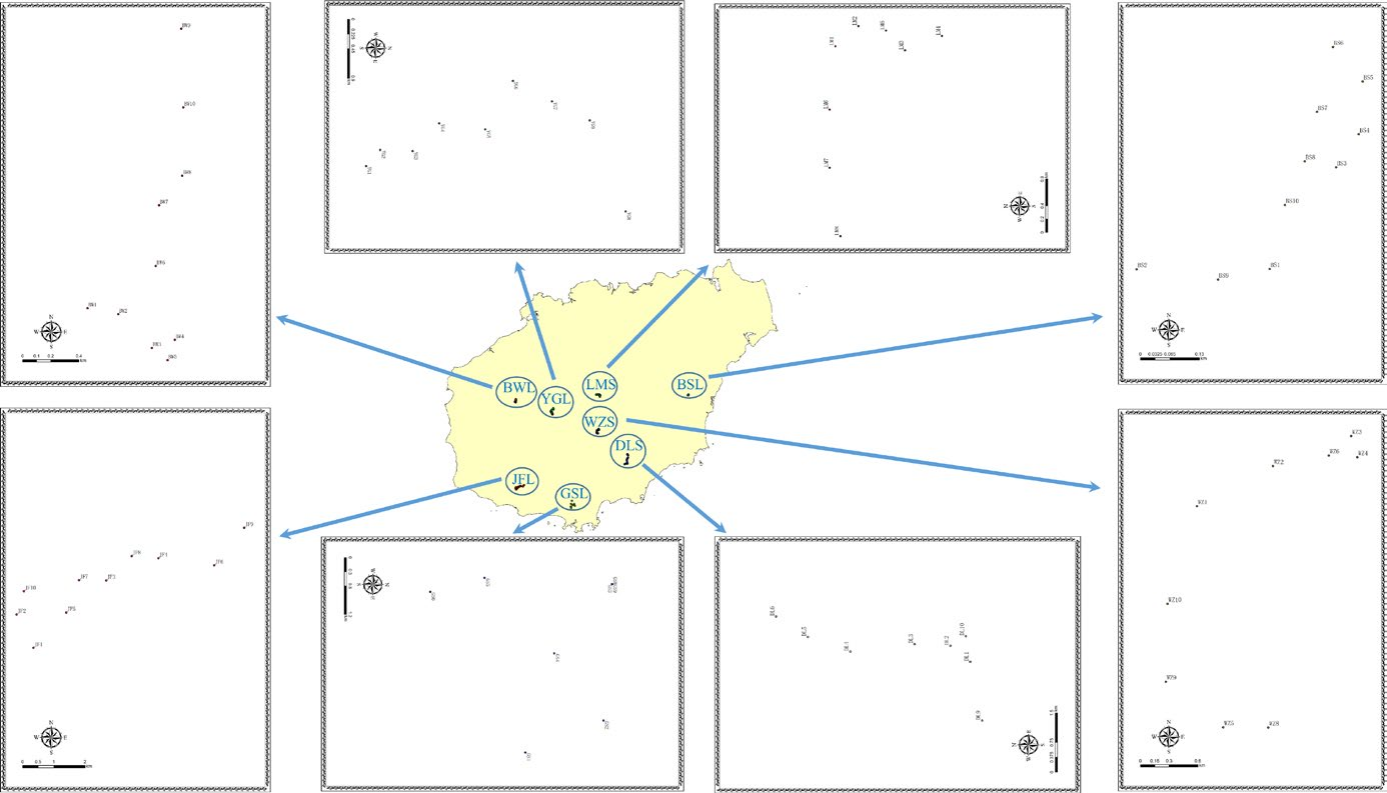
Distributions of sampling sites of eight populations of *Decalobanthus boisianus* in Hainan Island, China. BSL: population sampled from Baishiling Nature Reserve; BWL: population sampled from Bawangling National Nature Reserve; DLS: population sampled from Diaoluoshan National Nature Reserve; GSL: population sampled from Ganshiling Nature Reserve; JFL: population sampled from Jianfengling National Nature Reserve; LMS: population sampled from Limushan Nature Reserve; WZS: population sampled from Wuzhishan National Nature Reserve; YGL: population sampled from Yinggeling National Nature Reserve

The eight forests are located in Wuzhishan National Nature Reserve (WZS), Bawangling National Nature Reserve (BWL), Yinggeling National Nature Reserve (YGL), Diaoluoshan National Nature Reserve (DLS), Jianfengling National Nature Reserve (JFL), Limushan Nature Reserve (LMS), Baishiling Nature Reserve (BSL), and Ganshiling Nature Reserve (GSL) (Figure 1). We took the proportion of natural primary forest area occupying the whole land area in each region as a measure of the degree of human disturbance, and assumed that the greater proportion of natural primary forest means the smaller degree of human disturbance, and vice versa (FAO, 2005 2005; Sabatini et al., 2018) (Table 1). In light of the human disturbance intensity, we categorized *D. boisianus* in these eight forests into three groups, including low disturbance intensity group (WZS), moderate disturbance intensity group (YGL, BWL, JFL, and DLS), and high disturbance intensity group (LMS, BSL, and GSL).

**Table 1:** Site conditions of the eight *Decalobanthus boisianus* populations

Group	Code	Population	Sample size	Longitude (°E)	Latitude (°N)	Altitude (m)	Proportion of natural primary forests (%)
A	WZS	Wuzhishan National Nature Reserve, Wuzhishan County	10	109°37′–109°41′	18°43′–18°54′	300–800	54.20
	YGL	Yinggeling National Nature Reserve, Baisha County	10	109°21′–109°24′	19°01′–19°04′	300–500	14.10
	BWL	Bawangling National Nature Reserve, Changjiang County	10	109°02′–109°05′	19°07′–19°11′	150–600	13.50
B	JFL	Jianfengling National Nature Reserve, Ledong County	10	108°47′–108°49′	18°40′–18°43′	200–650	18.50
	DLS	Diaoluoshan National Nature Reserve, Lingshui County	10	109°53′–109°58′	18°43′–18°52′	100–600	12.60
	LMS	Limushan Nature Reserve, Qiongzhong County	8	109°42′–109°43′	19°09′–19°10′	500–750	0
C	GSL	Ganshiling Nature Reserve, Sanya City	9	109°38′–109°40′	18°22′–18°23′	100–300	0
	BSL	Baishiling Nature Reserve, Qionghai County	10	110°22′–110°23′	19°09′–19°10′	100–300	0

### 2.2 DNA extraction and PCR amplification

DNA was extracted from our 77 samples using the Plant DNA Isolation Kit (Foregene Co., Ltd., Chengdu, China). We followed the manufacturer's protocol using 25–30 mg of dried leaf material. DNA quality and quantity were determined visually by comparisons with the DNA marker DL2000 on 1.0% (W/V) agarose gel electrophoresis. Samples were then stored at −20°C prior to PCR amplification.

A set of 100 ISSR primers was synthesized by SinoGenoMax Co., Ltd. according to the sequences obtained from the University of British Columbia (Biotechnology Laboratory, University of British Columbia, primer set #9: http://www.biotech.ubc.ca/services/naps/primers/Primers.pdf). An initial experiment was performed to determine the suitable primer and reaction conditions. One sample randomly selected from each eight *D. boisianus* populations to test the preliminary number of polymorphic loci with 100 primers and to optimize the reaction and amplification procedure for PCR. Finally, we obtained optimal reaction and amplification procedures as well as 13 primers that produced a high number of variable and readable loci (Table 2).

**Table 2:** Primers selected from the UBC (University of British Columbia) used for ISSR amplification

Primers	Sequence	Annealing temperature (°C)	Fragment size (bp)	Loci number	Polymorphic loci number	Proportion of polymorphic loci (%)
UBC825	(AC)_8_T	52	200–1,300	15	13	87
UBC826	(AC)_8_C	52	200–1,500	19	18	95
UBC827	(AC)_8_G	52	150–1,300	19	19	100
UBC836	(AG)_8_YA	52	150–1,800	17	13	76
UBC841	(GA)_8_YC	52	150–1,200	16	12	75
UBC842	(GA)_8_YG	52	150–1,200	17	15	88
UBC849	(GT)_8_YA	52	200–1,800	12	12	100
UBC851	(GA)_8_YG	52	300–1,800	17	15	88
UBC856	(AC)_8_YA	54	200–1,300	18	14	78
UBC880	(GGAGA)_3_	54	150–1,300	19	15	79
UBC888	BDB(CA)_7_	54	150–1,100	17	15	88
UBC891	HVH(TG)_7_	54	200–1,400	19	15	79
UBC899	CAT(GT)_3_CATTGTTCCA	54	200–1,100	15	15	100

The PCR amplification was performed using the GeneAmp PCR System 9700 (Applied Biosystems, Carlsbad, CA, USA) in 20 μl reaction volumes containing 2 μl of DNA (50 ng/μl), 2 μl of primer (10 μM), 6 μl of ddH_2_O, and 10 μl of 1X Taq‐Plus PCR Forest Mix (included 0.1 U/μl Taq‐Plus DNA Polymerase Forest Reaction Buffer, 1.5 mM MgCl2, 200 μM of each dNTP). Polymerase chain reaction started with an initial activation step at 94°C for 4 min, followed by 45 cycles of denaturation at 94°C for 45 s, annealing at 52–54°C for 50 s (depending on primer, Table 2), extension at 72°C for 60 s, and then a final extension at 72°C for 6 min, followed by cooling at 4°C until recovery of the samples (Table 2).

The amplified products were separated on a 1.5% (W/V) agarose gel in 1× TAE buffer staining by GoldView (Beijing Solarbio Science & Technology Co., Ltd, Beijing, China) with a setup of 100V, 200 mA and 50 min and then visualized and photographed under ultraviolet light. A 2000 bp DNA ladder (Real times Biotechnology Co. Ltd., Beijing, China) was used to estimate the molecular weights in the Alphalmager—2200 UV Transilluminator (NatureGene Corp., Beijing, China).

### 2.3 Data analysis

Only bands that were clear and reproducible were used to construct data matrices. The amplified bands were scored as presence (1) or absence (0) of a particular band for each primer. These data were then used to assemble the data matrix of ISSR phenotypes.

### 2.4 The genetic diversity of the eight *D. boisianus* populations

We calculated the proportion of polymorphic loci (PPL), Shannon's diversity index (I), Nei's gene index (H), the observed number of alleles (Na), and the effective number of alleles (Ne) to assess levels of genetic diversity among our populations using the POPGENE program v. 1.32 (Francis, Rong, & Boyle, 1999). The PPL is an important indicator of genetic diversity and of the adaptability of populations to a particular environment. Shannon's index is based on the band phenotypic frequency and is used to estimate the genetic diversity within and between populations according to King and Schaal's method (King & Schaal, 1989). H is an index to reflect the gene diversity based on Hardy–Weinberg hypothesis (Nei, 1972). Other indices such as Na and Ne indicate the observed and effective numbers of alleles that are maintained in the populations (Johnson, 1974). Here, we compared the differences in genetic diversity of the eight *D. boisianus* populations across different human disturbance intensity using ANOVA analysis.

### 2.5 The genetic structure of the eight *D. boisianus* populations

Genetic differentiation among population (Gst) represents the portion of the total genetic diversity found among populations (Nei, 1972), and the number of migrants (gene flow, Nm) measures the degree of genetic differentiation among populations (Wright, 1950). Here, Gst, Nm, total genetic diversity (Ht), and gene diversity within populations (Hs) were used to determine the genetic differentiation among the eight populations of *D. boisianus*. A matrix of genetic similarity and Nei's genetic distances were used to depict genetic relationship among the eight *D. boisianus* populations (Nei, 1972). Populations with high genetic similarity and low Nei's genetic distances value indicate a close genetic relationship. All parameters were calculated using the POPGENE program v. 1.32 (Francis et al., 1999).

We also explored spatial structures of *D. boisianus* with a Bayesian model‐based cluster analysis, using the STRUCTURE program version 2.3.4 (Pritchard, Stephens, & Donnelly, 2000) and calculated the appropriate number of clusters of populations (K) (Evanno, Rengaut, & Gouget, 2005). Groups were chosen that best fit the genetic variability observed in the dataset, irrespective of the number of populations sampled (Andreakis, Kooistra, & Procaccini, 2009). After initial pilot runs of variable burn‐in and run‐length, 20 independent runs were performed at K = 2–8 with 10,000 MCMC repetitions and a burn‐in period of 10,000 iterations, using no prior information and assuming correlated allele frequencies and admixture model in this study. The STRUCTURE output was further interpreted by STRUCTURE HARVESTE (Earl & VonHoldt, 2012). The CLUMPP version 1.1 (Jakobsson & Rosenberg, 2007) were used with greedy algorithms, with 1,000 random input orders and 1,000 repeats to calculate the average pairwise similarity of runs. The clustered output was visualized using the software Distruct version 1.1 (Rosenberg, 2004). Finally, we conducted cluster analysis for the eight populations with the MEGA v.10.0.1 (Kumar, Stecher, Li, Knyaz, & Tamura, 2018) based on the Nei's genetic distances and bootstrap analysis with 5,000 replications using the unweighted pair‐group method of averages (UPGMA).

Geographic distances (in kilometers) among populations were estimated by using toolbox in ArcGIS 10.3 (Esri: California, CA, USA) according to the coordinate (the longitude and latitude) of each population. A Mantel test was performed to assess isolation‐by‐distance with 1,000 random permutations, to test the significance of the relationship between ɸst and geographic distances. The distribution of whole genetic diversity was estimated using the molecular variance (AMOVA), taking populations and individuals as different levels. The significance of ɸst genetic differentiation was assessed with 1,000 random permutations. The principal coordinate analysis (PCoA) was carried out for the genetic relationships among populations. These analyses above (Mantel test, AMOVA, and PCoA) were carried out using the GenALEx6.5 program (Peakall & Smouse, 2012).

## 3 RESULTS

### 3.1 PCR amplification

A total of 220 unique loci ranging in size from 150 to 1800 bp were scored from ISSR marker products. From these, 198 (90.00%) were polymorphic (Table 3). A mean of 18 loci was generated per primer, in which the number of loci varied from 13 to 21. The UBC827, UBC849, and UBC899 primers had the highest proportion of polymorphic loci (100%) per *D. boisianus* population, whereas UBC841 had the lowest one (75%).

**Table 3:** Genetic diversity of eight populations of *Decalobanthus boisianus* based on 220 ISSR loci

Population	Polymorphic loci	PPL (%)	Na	Ne	I	H
YGL	126	57.27	1.57	1.24	0.24	0.15
BWL	128	58.18	1.58	1.29	0.27	0.17
JFL	94	42.73	1.43	1.24	0.21	0.14
GSL	76	34.55	1.35	1.20	0.17	0.11
LMS	87	39.55	1.40	1.22	0.20	0.13
WZS	146	66.36	1.66	1.33	0.31	0.20
DLS	110	50.00	1.50	1.24	0.22	0.14
BSL	81	36.82	1.37	1.22	0.19	0.13
Mean (with ± *SD*)	106	48.18	1.48 (±0.11)	1.25 (±0.04)	0.23 (±0.04)	0.15 (±0.03)
Species level (with ± *SD*)	198	90.00	2.00 (±0.00)	1.33 (±0.33)	0.33 (±0.22)	0.21 (±0.17)

Note

H: Nei's gene index; I: Shannon's diversity index; Na: the observed number of alleles; Ne: the effective number of alleles; PPL: the proportion of polymorphic loci.

### 3.2 Genetic diversity

Among the eight *D. boisianus* populations collecting from the eight forest sites, the mean values of the proportion of polymorphic loci, the observed number of alleles (Na), the effective number of alleles (Ne), Shannon's diversity index (I), and Nei's gene index (H) were 48.18%, 1.48 ± 0.11, 1.25 ± 0.04, 0.23 ± 0.04, and 0.15 ± 0.03, respectively (Table 3). At the species level, PPL, Na, Ne, I, and H were 90.00%, 2.00 ± 0.00, 1.33 ± 0.33, 0.33 ± 0.22, and 0.20 ± 0.17, respectively (Table 3).

We also found the genetic diversity varies significantly with human disturbance intensity, with the high, medium, and low genetic diversity observing in low‐intensity group (*D. boisianus *population in WZS), moderate intensity group (*D. boisianus* population in YGL, BWL, JFL, DLS), and high‐intensity group (*D. boisianus *populatoin in LMS, BSL and GSL), respectively (Polymorphic loci: *F*_(2,8)_ = 18.89, *p* = 0.003; PPL: *F*_(2,8)_ = 18.89, *p* = 0.003; Na: *F*_(2,8)_ = 19.08, *p* = 0.003; Ne: *F*_(2,8)_ = 23.05, *p* = 0.002; I: *F*_(2,8)_ = 21.34, *p* = 0.002; and H: *F*_(2,8)_ = 24.54, *p* = 0.001).

### 3.3 Genetic structure

Among the eight populations, the total gene diversity index (H_t_) and gene diversity within population index (H_s_) were 0.21 ± 0.03 and 0.15 ± 0.01, respectively. The genetic differentiation index among the eight populations (Gst) was 0.28. AMOVA analysis showed that a relatively large proportion of genetic variation (75.67%) occurred within the eight *D. boisianus *populations, whereas only 24.33% of genetic variation was observed among the eight populations (ɸst = 0.24, *p* < 0.001; Table 4). The historical mean number of migrants (Nm) was 1.27, indicating the effective gene flow among the eight *D. boisianus* populations.

**Table 4:** Analysis of molecular variance (AMOVA) for 8 populations of *Decalobanthus boisianus *based on 220 ISSR loci

Source of variation	*df*	SSD	MSD	Variance component	Total variance (%)	*p* *
Among populations	7	574.09	82.01	6.45	24.33	<0.001
Within populations	69	1382.71	20.04	20.04	75.67	<0.001
Total	76	1956.81	102.05	26.48	100	

Note

*df*, the degree of freedom; SSD, the sum of squared deviations; MSD, the mean squared deviations.

* Indicated significance tests after 1,000 permutations.

The mean value of genetic similarity among the eight *D. boisianus* populations was high (0.93), while the genetic distances among the eight *D. boisianus* populations were relative low (0.07), indicating the close relationships among the eight *D. boisianus* populations (Table S1). The Mantel test's results (*r* = 0.39, *p* = 0.10) indicated that there were no significant correlations between Nei's genetic distances and geographic distances.

**Figure 2:**
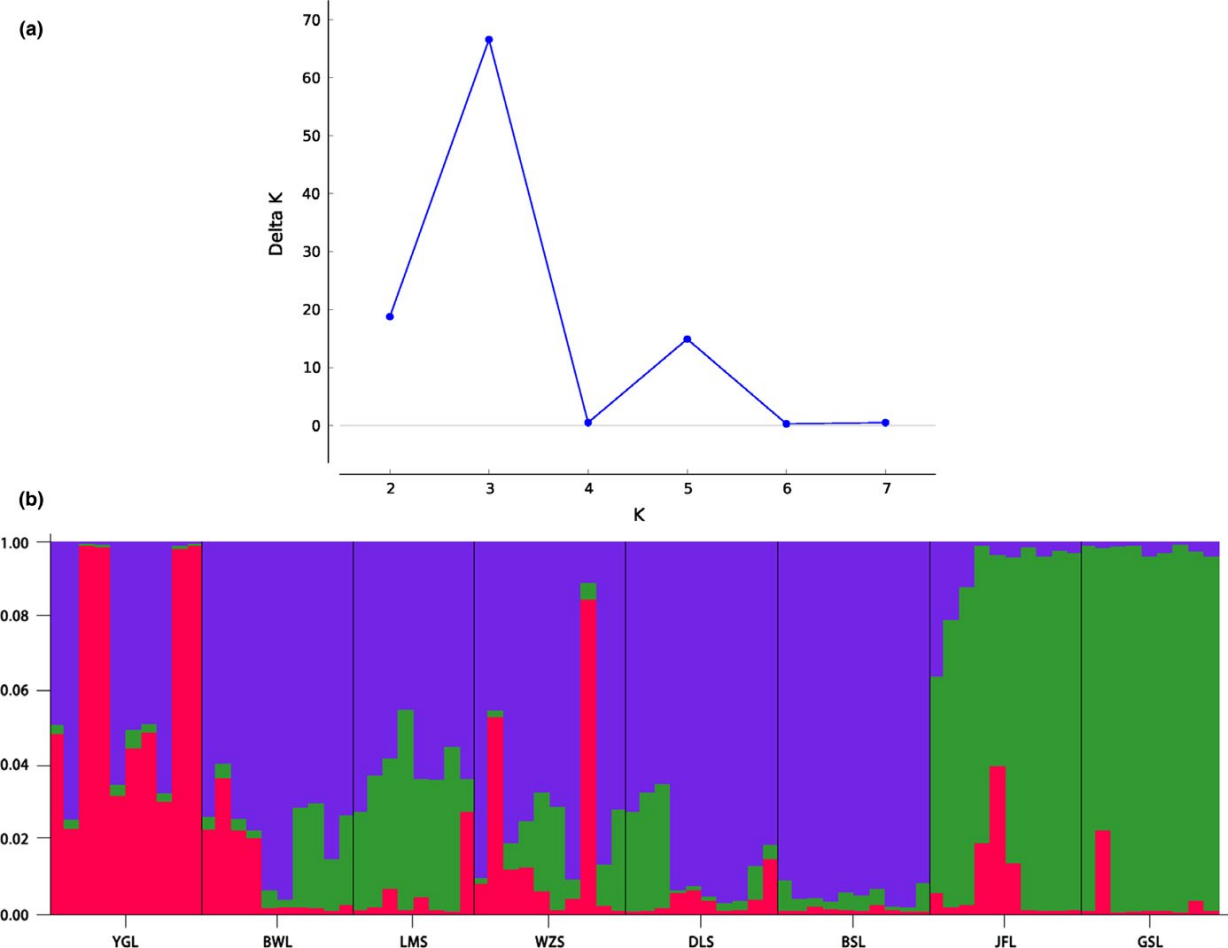
Cluster of 77 individuals from eight *Decalobanthus boisianus* populations using made by STRUCTURE for K = 3. Each individual is represented by a vertical, colored line. Same color in different individuals indicates that they are belonging to the same cluster. BSL: population sampled from Baishiling Nature Reserve; BWL: population sampled from Bawangling National Nature Reserve; DLS: population sampled from Diaoluoshan National Nature Reserve; GSL: population sampled from Ganshiling Nature Reserve ; JFL: population sampled from Jianfengling National Nature Reserve; LMS: population sampled from Limushan Nature Reserve; WZS: population sampled from Wuzhishan National Nature Reserve; YGL: population sampled from Yinggeling National Nature Reserve

**Figure 3:**
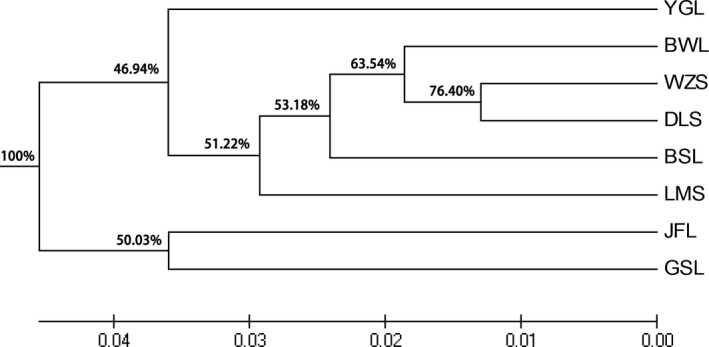
UPGMA dendrogram based on Nei's genetic distances among 8 populations of *Decalobanthus boisianus*. BSL: population sampled from Baishiling Nature Reserve; BWL: population sampled from Bawangling National Nature Reserve; DLS: population sampled from Diaoluoshan National Nature Reserve; GSL: population sampled from Ganshiling Nature Reserve; JFL: population sampled from Jianfengling National Nature Reserve; LMS: population sampled from Limushan Nature Reserve; WZS: population sampled from Wuzhishan National Nature Reserve; YGL: population sampled from Yinggeling National Nature Reserve

**Figure 4:**
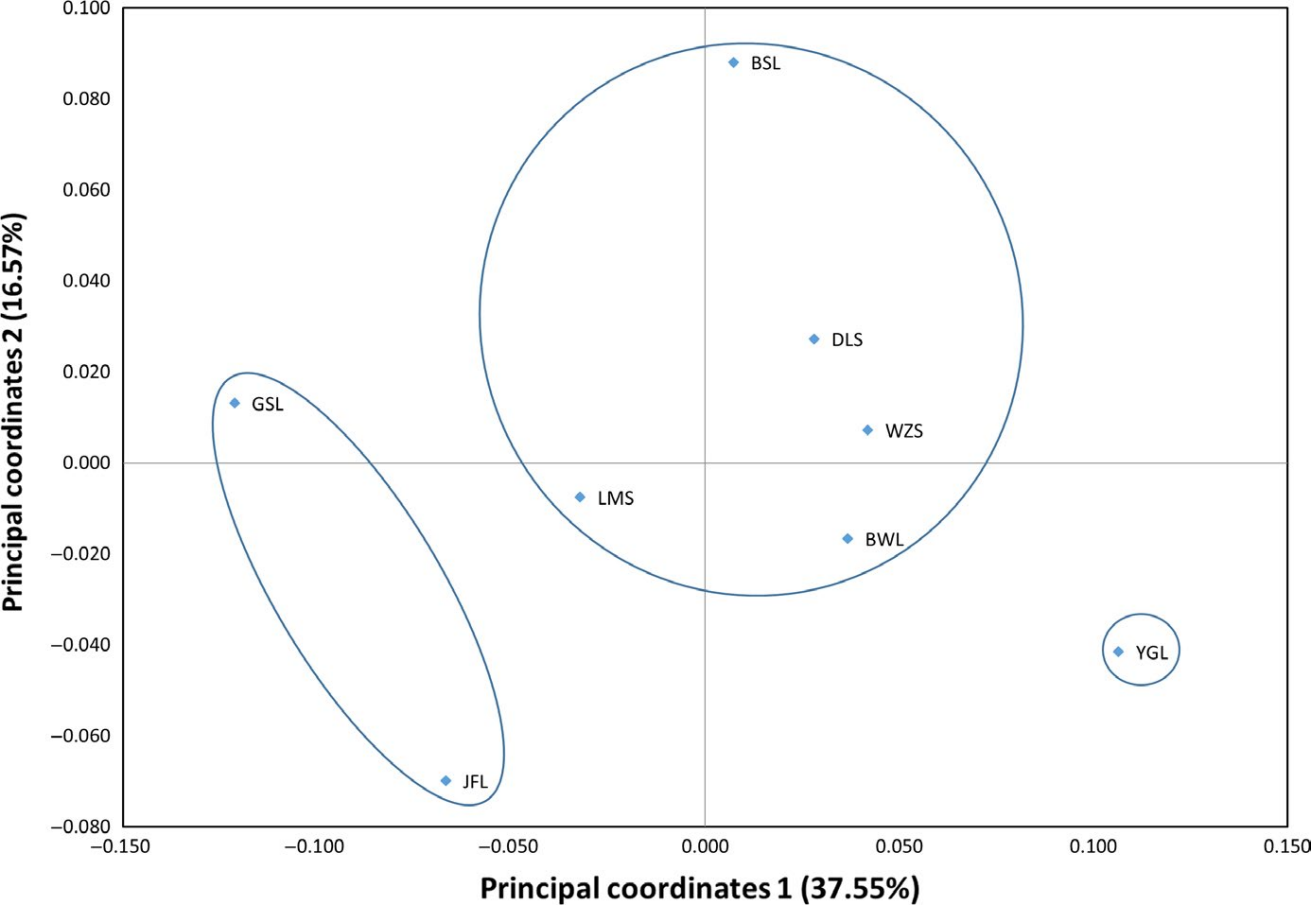
Principal coordinate analysis (PCoA) analyses showing the genetic structures of *Decalobanthus boisianus* populations. BSL: population sampled from Baishiling Nature Reserve; BWL: population sampled from Bawangling National Nature Reserve; DLS: population sampled from Diaoluoshan National Nature Reserve; GSL: population sampled from Ganshiling Nature Reserve; JFL: population sampled from Jianfengling National Nature Reserve; LMS: population sampled from Limushan Nature Reserve; WZS: population sampled from Wuzhishan National Nature Reserve; YGL: population sampled from Yinggeling National Nature Reserve

STRUCTURE analysis showed that delta K displayed two peaks (K = 3 and K = 5) but the maximum one was obtained at K = 3 (Figure 2a), suggesting the eight *D. boisianus* populations can be clustered into three groups, including group 1 (populations in BWL, WZS, DLS, BSL, and LMS), group 2 (populations in JFL and GSL) and group 3 (population in YGL). These three groups, moreover, were confirmed using UPGMA dendrogram (Figure 3) and PCoA analyses (Figure 4).

## 4 DISCUSSION

### 4.1 Genetic diversity of *D. boisianus* varies with human disturbance intensity

Genetic diversity of the eight *D. boisianus* populations significantly increase with the decrease in human disturbance intensity (Polymorphic loci: *F*_(2,8)_ = 18.89, *p* = 0.003; PPL: *F*_(2,8)_ = 18.89, *p* = 0.003; Na: *F*_(2,8)_ = 19.08, *p* = 0.003; Ne: *F*_(2,8)_ = 23.05, *p* = 0.002; I: *F*_(2,8)_ = 21.34, *p* = 0.002; H: *F*_(2,8)_ = 24.54, *p* = 0.001), which proves our hypotheses. Human disturbance may influence the balance between sexual and clonal reproduction of *D. boisianus* populations (Hamrick et al., 1979; Loveless & Hamrick, 1984), to alter genetic diversity of *D. boisianus* populations (Cook, 1985; Eriksson, 1997; Sinclair et al., 2016). For example, *D. boisianus* in LMS, BSL and GSL with high‐intensity human disturbances may allocate resources to clonal reproduction, which helps it rapidly expand to novel habitats. But the clonal reproduction produces the same offspring gene and leads to low genetic diversity (Ellstrand & Roose, 1987; Philbrick & Les, 1996; Kudoh, 1999). In contrast, low‐intensity human disturbances allow WZS population to allocate more resources to sexual reproduction, thereby maintaining higher level of genetic diversity (Prentis et al., 2008; Silander, 1979). Our results are consistent with Aveliina et al. (2009), which demonstrated that human activity leads to a decrease in genetic diversity for *Briza media*. Although our study shows the relationships between the genetic diversity of *D. Boisiana* and human disturbance intensity, the cause of the conversion between sexual and clonal reproduction still need large‐scale manipulative experiments.

### 4.2 Genetic structure of *D. boisianus* in Hainan Island

In our study, both Nei's genetic differentiation index (Gst = 0.28) and AMOVA values (24.88%) indicated that there was a large proportion of genetic variation (over 70%) present within the eight *D. boisianus *populations, while less than 30% of genetic variation was observed among the eight populations. These results are consistent with the previous reports from other invasive species like *Carduus acanthoides* L. (Bohumil, Petr, Dana, Petr, & Ivana, 2009), *Ambrosia artemisiifolia* L. (Kočiš et al., 2015), and *Spartina densiflora* Brongn. (Castillo et al., 2018) but are less than most clonal invasive species, such as *Eupatorium catarium *Veldkamp (Li, Li, & Liu, 2014), *Praxelis clematidea* R.M.King & H.Rob. (Wang, Huang, Downie, Chen, & Chen, 2015), *Galinsoga quadriradiata* Ruiz & Pav. (Li, Qi, Yao, & Liu, 2015), *Mikania micrantha* H.B.K. (Li, Dong, & Zhong, 2007), and *Alternanthera philoxeroides* Griseb. (Ye, Li, Cao, & Ge, 2003). These results indicate that a very low genetic differentiation of the eight *D. boisianus *populations is observed on Hainan Island. One possible reason is that the effective gene flow (1.27) among the eight populations result from the high dispersal ability of *D. boisianus* to against barriers throughout the expansion of this species (Opedal et al., 2017; Wright, 1950). Indeed, the small mean value of Nei's genetic distance (0.07) and the high mean genetic similarity index (0.93) both indicate the close relationship among *D. boisianus *populations. Although little is known for the dispersal mode of this species, many studies have assumed that viable seeds of *D. boisianus* may be transported over streams to achieve long‐distance transmission (Wu et al., 2007; Wang et al., 2009). Moreover, several species that are closely related to *D. boisianus*, such as *D. peltata*, *Merremia palmeri *Hallier f., and *Merremia ellenbeckii *Pilg, were found that could be transported over streams (Francisco & Clara, 1997; Taylor & Kumar, 2016; Thulin, 2010). Value for ɸst (0.24) of *D. boisianus* is very close to those of hydrophilous plants (0.25) (Nybom, 2004), too. Hence, this stream dispersal mode may therefore generate the effective gene flow across large distances for *D. boisianus*. On the other side, intense storms on Hainan Island can also increase opportunities for propagules dispersal of *D. boisianus* and move them long distances across the landscape, downstream, and across catchments (Diez et al., 2012, Murphy & Metcalfe et al., 2016), and therefore accelerated the movement and gene exchange frequency among *D. boisianus* populations. Therefore, we should try to limit the spread of seeds and propagules of this weed. Further research is needed to identify the optimal management to control this species, for example, introduction of host‐specific natural enemies and proper disposal of this species.

UPGMA dendrogram, Bayesian analysis and PCoA analysis all show that the eight *D. boisianu* populations are categorized to three different groups (Figures 2, 3, and 4), which is not match the three groups categorized by the human disturbance intensity (Table 1), suggest that human disturbance cannot affect genetic structure of *D. boisianu* populations. In spite of geographic distances is nonsignificant (Mantel test, *r* = 0.34, *p* = 0.09) with genetic distances of this species, some nearby populations have clustered together, such as WZS with LMS and BSL, JFL with GSL populations. This implied that the horizontal expansion of clones might also lead to gene flow among nearby populations of that species and made their genetic relationships closer.

## 5 CONCLUSION

Generally, we found that genetic diversity of *D. boisianus* was correlated to the human disturbance intensity, which has been proved to be a major factor governing plant invasion. High dispersal ability and adaptive potential of *D. boisianus* may be important factors that were attributed to its large distances expansion and cause serious damage to the ecosystems. So far, artificial removal and chemical controls have been implemented to kill and prevent *D. boisianus* in the study areas, but were seldom effective. We suggest reducing deforestation to decrease human disturbance to forests for one hand. In addition, effective efforts should be taken to remove *D. boisianus*, and restore the secondary forests.

## AUTHOR CONTRIBUTIONS

Huan Jiang, Wenxing Long, Hui Zhang, Chengneng Mi, Tao Zhou, and Zongzhu Chen conceived and designed the experiments; Huan Jiang and Wenxing Long performed the laboratory work; Hui Zhang and Zongzhu Chen analysed the data; Huan Jiang, Wenxing Long, Hui Zhang, and Zongzhu Chen wrote the paper.

## Data Availability

Inter Simple Sequence Repeat (ISSR) data for *Decalobanthus boisianus* is available from the Dryad Digital Repository: https://doi.org/10.5061/dryad.7nq1636.
