# Supplementary material for: Genetic diversity and genetic structure of Decalobanthus boisianus in Hainan Island, China
Source: Ecol Evol. 2019 Apr 18;9(9):5362–71. doi: 10.1002/ece3.5127 (PMC6509374; doi:10.1002/ece3.5127)
Supplement: Supplementary file 1 [file ECE3-9-5362-s001.doc]

**Supporting Information**

**Genetic diversity and genetic structure of *Decalobanthus boisianus* in Hainan Island, China**

Huan Jiang^1,2^, Wenxing Long^1,2*^, Hui Zhang^1,2*^, Chengneng Mi^1,2^, Tao Zhou^1,2^, Zongzhu Chen^3^

^1^ Institute of Tropical Agriculture and Forestry, Hainan University, Haikou 570228, China.

^2^ Wuzhishan National Long Term Forest Ecosystem Research Station, Hainan, 570000, China.

^3^ Institute of Forestry Science of Hainan Province, Haikou 570000, China.

* Both authors were listed as correspondence: Wenxing Long, e-mail: oklong@hainu.edu.cn

Table S1 Genetic similarity and genetic distance between 8 populations of *Decalobanthus boisianus* based on 220 ISSR loci.

| Population | YGL | BWL | LMS | WZS | DLS | BSL | JFL | GSL |
| --- | --- | --- | --- | --- | --- | --- | --- | --- |
| YGL | － | 0.94 | 0.91 | 0.96 | 0.94 | 0.91 | 0.89 | 0.86 |
| BWL | 0.06 | － | 0.94 | 0.96 | 0.96 | 0.94 | 0.93 | 0.90 |
| LMS | 0.10 | 0.06 | － | 0.96 | 0.94 | 0.93 | 0.93 | 0.93 |
| WZS | 0.04 | 0.04 | 0.05 | － | 0.97 | 0.96 | 0.93 | 0.91 |
| DLS | 0.07 | 0.03 | 0.06 | 0.03 | － | 0.96 | 0.94 | 0.91 |
| BSL | 0.09 | 0.06 | 0.07 | 0.05 | 0.04 | － | 0.92 | 0.92 |
| JFL | 0.12 | 0.07 | 0.07 | 0.07 | 0.07 | 0.09 | － | 0.93 |
| GSL | 0.15 | 0.10 | 0.07 | 0.09 | 0.10 | 0.09 | 0.07 | － |

Nei's genetic similarity is above diagonal and genetic distance is below diagonal.
